# A simplified curcumin targets the membrane of *Bacillus subtilis*

**DOI:** 10.1002/mbo3.683

**Published:** 2018-07-26

**Authors:** Luana G. Morão, Carlos R. Polaquini, Malgorzata Kopacz, Guilherme S. Torrezan, Gabriela M. Ayusso, Guilherme Dilarri, Lúcia B. Cavalca, Aleksandra Zielińska, Dirk‐Jan Scheffers, Luis O. Regasini, Henrique Ferreira

**Affiliations:** ^1^ Departamento de Bioquímica e Microbiologia Instituto de Biociências Universidade Estadual Paulista Rio Claro Brazil; ^2^ Departamento de Química e Ciências Ambientais Instituto de Biociências Letras e Ciências Exatas Universidade Estadual Paulista São José do Rio Preto Brazil; ^3^ Department of Molecular Microbiology Groningen Biomolecular Sciences and Biotechnology Institute University of Groningen Groningen The Netherlands

**Keywords:** antibacterial compound, cell division, membrane permeabilization, turmeric

## Abstract

Curcumin is the main constituent of turmeric, a seasoning popularized around the world with Indian cuisine. Among the benefits attributed to curcumin are anti‐inflammatory, antimicrobial, antitumoral, and chemopreventive effects. Besides, curcumin inhibits the growth of the gram‐positive bacterium *Bacillus subtilis*. The anti‐*B. subtilis* action happens by interference with the division protein FtsZ, an ancestral tubulin widespread in *Bacteria*. FtsZ forms protofilaments in a GTP‐dependent manner, with the concomitant recruitment of essential factors to operate cell division. By stimulating the GTPase activity of FtsZ, curcumin destabilizes its function. Recently, curcumin was shown to promote membrane permeabilization in *B. subtilis*. Here, we used molecular simplification to dissect the functionalities of curcumin. A simplified form*,* in which a monocarbonyl group substituted the β‐diketone moiety, showed antibacterial action against gram‐positive and gram‐negative bacteria of clinical interest. The simplified curcumin also disrupted the divisional septum of *B. subtilis*; however, subsequent biochemical analysis did not support a direct action on FtsZ. Our results suggest that the simplified curcumin exerted its function mainly through membrane permeabilization, with disruption of the membrane potential necessary for FtsZ intra‐cellular localization. Finally, we show here experimental evidence for the requirement of the β‐diketone group of curcumin for its interaction with FtsZ.

## 1 INTRODUCTION

Molecular simplification is a strategy used for the discovery and development of new drugs based on bioactive natural products exhibiting complex molecular structures or toxic groups. The concept of molecular simplification is related to the withdrawal of functional groups from prototype molecules, while keeping or enhancing desirable bioactivity. Lidocaine, for example, is a simplified form of cocaine that retains the local anesthetic properties without the narcotic side effects (Gordh, 2010). Phenetidine is a simplified morphine, which has demonstrated similar analgesic activity (Janssen & van der Eycken, 1968). The molecular simplified analogue of quinine, mefloquine, exhibits potent antimalarial action, and is also effective against resistant strains of *Plasmodium* (Grigg et al., 2016; Murray & Perkins, 1996). Finally, the analogue designed by the molecular simplification of piperlongumine, a natural alkaloid, is more active and selectively cytotoxic against breast cancer cells than the prototype molecule (Valli et al., 2017).

Curcumin (1) is a diphenolic compound extracted from the rhizomes of *Curcuma longa*, which exhibits a large range of bioactive properties that include antifungal, antiviral, and anticancer activities (Chen et al., 2016; Di Martino et al., 2017; Kumar et al., 2014). The antibacterial activity of curcumin and its natural and synthetic analogues has been extensively revised by Moghadamtousi et al. (2014). However, our main interest in curcumin relates to its ability to inhibit bacterial cytokinesis (Kaur, Modi, Panda, & Roy, 2010; Rai, Singh, Roy, & Panda, 2008). Division in bacterial cells requires the proper assembly of a septal structure, the Z‐ring, which has as main constituent the tubulin‐like protein FtsZ (Lutkenhaus, 2007). FtsZ has a pivotal function during this process, as it acts as a division scaffold and recruits several other cellular factors that assemble the divisome (the protein complex operating on cell division) (Hurley et al., 2016; Xiao & Goley, 2016). FtsZ polymerizes in a GTP‐dependent manner into protofilaments that associate to compose the Z‐ring at the site of division. Curcumin was shown to inhibit the assembly of FtsZ protofilaments in *Bacillus subtilis* (*B. subtilis*), where it exerts its function by stimulating the GTPase activity of FtsZ (Rai et al., 2008).

**Figure 1:**
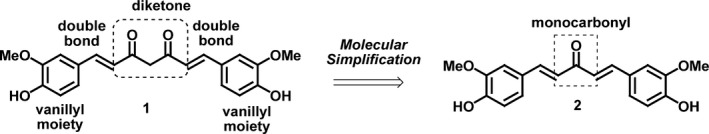
Curcumin (1) and its simplified analogue (2)

Another function that has been attributed to curcumin is the ability to modify lipid bilayers, which may consequently alter the function of membrane proteins (Hung et al., 2008; Ingolfsson, Koeppe, & Andersen, 2007). Supporting this, curcumin I, the main constituent of the seasoning turmeric, was found to permeabilize bacterial membranes (Tyagi, Singh, Kumari, Kumari, & Mukhopadhyay, 2015). The pool of biological properties of curcumin has been recognized as pan‐assay interference compound (PAIN) and invalid metabolic panacea (IMP) (Nelson et al., 2017). These classifications are attributed to prototypes that exhibit poor performance as lead compounds due to their activities in various biological assays in which they may interfere with the assay reading rather than displaying a specific interaction with a target. Among the functionalities of the curcumin structure, the β‐diketone moiety seems to be related to the PAIN and IMP behaviors, especially by metal chelation and structural decomposition (Hampannavar, Karpoormath, Palkar, & Shaikh, 2016; Heger, van Golen, Broekgaarden, & Michel, 2014). Taking this into account, we designed and synthesized a simplified analogue of curcumin (2) in which a monocarbonyl group replaced the β‐diketone, while preserving both double bonds, as well as the vanillyl moieties (Figure 1). The antibacterial activity of 2 and its analogues (compounds 3–8) were evaluated against a panel of gram‐positive and gram‐negative bacteria. Compound 2 was active against the plant pathogen *Xanthomonas citri* (*X. citri*), the clinically relevant strains *Salmonella enterica* sorovar Typhimurium (*S. enterica*) and *Enterococcus faecalis* (*E. faecalis*), and the gram‐positive bacterial model *Bacillus subtilis* (*B. subtilis*). Moreover, 2 was able to disrupt the divisome of *B. subtilis*; however, further biochemical analysis did not support a direct and/or specific action on FtsZ. Finally, our data support the view that 2 exerts its function against *B. subtilis* by membrane pore formation, which suggests that the β‐diketone moiety is required for the interaction of curcumin and FtsZ.

## 2 RESULTS

### 2.1 Synthesis and ^1^H NMR spectrum data of simplified curcumin (2) and its analogues (3–8)

**Figure 2:**
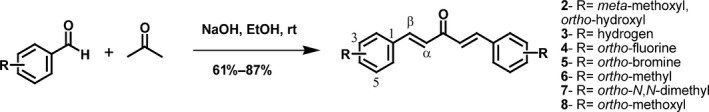
Synthesis of simplified curcumin (2) and its analogues (3–8)

Synthesis of 2 and its analogues (3–8) was achieved by the reaction between the respective benzoic aldehydes and acetone. Analogues of 2 bearing phenyl rings symmetrically substituted by halogens (–F and –Br) and electron‐donating [–CH_3_, –N(CH_3_)_2_ and –OCH_3_] substituents were synthesized, with yields ranging from 61% to 87% (Figure 2). The structure of 2–8 was identified by ^1^H NMR spectrum data analyses. The most characteristic signals observed in the ^1^H NMR spectra were those of the olefinic double bond hydrogen, which were present as a pair of doublets with coupling constants ranging from 15.6 to 16.2 Hz, indicating the *trans* configuration. For all the compounds, NMR parameters corresponded with the proposed structures.

(1*E*,4*E*)‐1,5‐bis(4‐hydroxy‐3‐methoxyphenyl)penta‐1,4‐dien‐3‐one (2): Yellow solid. 68% yield. ^1^H NMR (600 MHz; CDCl_3_) δ_H_ (mult.; *J* in Hz): 7.70 (d; 16.2; H‐β), 7.21 (dd; 2.4 and 8.4; H‐6), 7.14 (d; 2.4; H‐2), 6.97 (d; 8.4; H‐5), 6.96 (d; 16.2; H‐α), 5.95 (br s; 4 – OH), 3.98 (s; 3 – OCH_3_).

(1*E*,4*E*)‐1,5‐diphenylpenta‐1,4‐dien‐3‐one (3): Yellow solid. 61% yield. ^1^H NMR (600 MHz; CDCl_3_) δ_H_ (mult.; *J* in Hz): 7.77 (d; 15.6; H‐β), 7.65 (dd; 1.8 and 6.6; H‐2 and H‐6), 7.45 (m; H‐3, H‐4 and H‐5), 7.12 (d; 15.6; H‐α).

(1*E*,4*E*)‐1,5‐bis(4‐fluorophenyl)penta‐1,4‐dien‐3‐one (4): Yellow solid. 84% yield. ^1^H NMR (600 MHz; CDCl_3_) δ_H_ (mult.; *J* in Hz): 7.73 (d; 15.6; H‐β), 7.64 (d; 8.4; H‐2 and H‐6), 7.14 (dd; 8.4 and 9.0; H‐3 and H‐5), 7.02 (d; 15.6; H‐α).

(1*E*,4*E*)‐1,5‐bis(4‐bromophenyl)penta‐1,4‐dien‐3‐one (5): Yellow solid. 75% yield. ^1^H NMR (600 MHz; CDCl_3_) δ_H_ (mult.; *J* in Hz): 7.69 (d; 15.6; H‐ β), 7.58 (d; 8.4; H‐2 and H‐6), 7.50 (d; 8.4; H‐3 and H‐5), 7.07 (d; 15.6; H‐α).

(1*E*,4*E*)‐1,5‐bis(4‐methylphenyl)penta‐1,4‐dien‐3‐one (6): Yellow solid. 63% yield. ^1^H NMR (600 MHz; CDCl_3_) δ_H_ (mult.; *J* in Hz): 7.74 (d; 16.2; H‐ β), 7.55 (d; 8.4; H‐2 and H‐6), 7.25 (d; 8.4; H‐3 and H‐5), 7.07 (d; 16.2; H‐ α), 2.42 (s; 4 – CH_3_).

(1*E*,4*E*)‐1,5‐bis(4‐(dimethylamino)phenyl)penta‐1,4‐dien‐3‐one (7): Red solid. 87% yield. ^1^H NMR (600 MHz; CDCl_3_) δ_H_ (mult.; *J* in Hz): 7.71 (d; 16.2; H‐ β), 7.54 (d; 9.0; H‐2 and H‐6), 6.92 (d; 16.2, H‐ α), 6.72 (d; 9.0; H‐3 and H‐5), 3.06 (s; 4 – N(CH_3_)_2_).

(1*E*,4*E*)‐1,5‐bis(4‐methoxyphenyl)penta‐1,4‐dien‐3‐one (8): Yellow solid. 74% yield. ^1^H NMR (600 MHz; CDCl_3_) δ_H_ (mult.; *J* in Hz): 7.73 (d; 16.2; H‐ β), 7.60 (d; 8.4; H‐2 and H‐6), 6.98 (d; 16.2; H‐ α), 6.96 (d; 8.4; H‐3 and H‐5), 3.88 (s; 4 – OCH_3_).

### 2.2 Preliminary screening of the antibacterial activity of 2 and its analogues

The antibacterial activities of the compounds 1 (curcumin) and 2–8 were evaluated against a panel of gram‐positive and gram‐negative bacteria using *resazurin* microtiter assay plate (REMA), an assay that measures the cell respiratory activity by the indirect detection of NADH production. In order to guarantee a fair comparison among the tests (number of cells exposed/concentration of compound), the amount of cells was standardized to 10^5^ and the concentration of each of the compounds under analysis was fixed at 100 μg/mL. Curcumin (1) was active against six of seven bacteria tested, except for the plant pathogen *X. citri*. The percentage of growth inhibition caused by 1 for those affected varied from 52% to 98% (Table 1; growth inhibition was determined as a diminished percentage of resazurin reduction to its fluorescent form resorufin when comparing untreated and treated cells). Besides, 1 was more active against the gram‐positive strains *S. aureus* and *B. subtilis*, reaching inhibition levels of more than 90% at the concentration of 100 μg/mL. When we consider the simplified forms derived from 1, two important human pathogens, *S. enterica* and *E. faecalis*, were the preferred targets. *S. enterica* was the most sensitive strain being inhibited by all of the compounds tested (2–8) with percentages of inhibition varying from ~60% to 75%. Next, *E. faecalis* was inhibited by four of seven compounds (2, 3, 7, and 8) and displayed a growth inhibition ranging from 62%–78%. Noteworthy, compound 2 was the only one to inhibit the phytopathogen *X. citri*, the etiological agent of citrus canker, which is a severe disease that affects citrus plants worldwide. Compound 2 was active against four different species, two gram‐negative and two gram‐positive, considering the inhibition level of ~60% or above. Additionally, 2 inhibited growth of nearly 80% of *E. faecalis*, an important human pathogen with documented resistance to diverse antibiotics, and more than 90% of *B. subtilis*. Therefore, compound 2 was chosen for further characterization.

**Table 1:** Antibacterial activity of simplified curcumin and its analogues

	Bacteria	Compound (100 μg/mL) Percentage of growth inhibition^a^							
		1	2	3	4	5	6	7	8
Gram‐negative	*Xanthomonas citri* IBSBF‐1594	<10	61	<10	<10	<10	34	47	<10
	*Pseudomonas aeruginosa* ATCC27853	67	<10	<10	<10	<10	<10	<10	<10
	*Salmonella enterica* sorovar Typhimurium ATCC13311	66	59	67	72	61	70	75	73
	*Escherichia coli* ATCC25922	65	<10	<10	<10	<10	<10	38	<10
Gram‐positive	*Staphylococcus aureus* ATCC25923	95	21	<10	22	12	<10	11	<10
	*Enterococcus faecalis* ATCC29212	52	78	62	21	<10	44	68	70
	*Bacillus subtilis* Strain 168	98	93	78	35	18	<10	<10	19

a Growth inhibition is inversely proportional to the amount of resazurin reduced by NADH to resorufin, where 100% reduction means no growth inhibition.

### 2.3 Activity of 2 against *B. subtilis*

**Figure 3:**
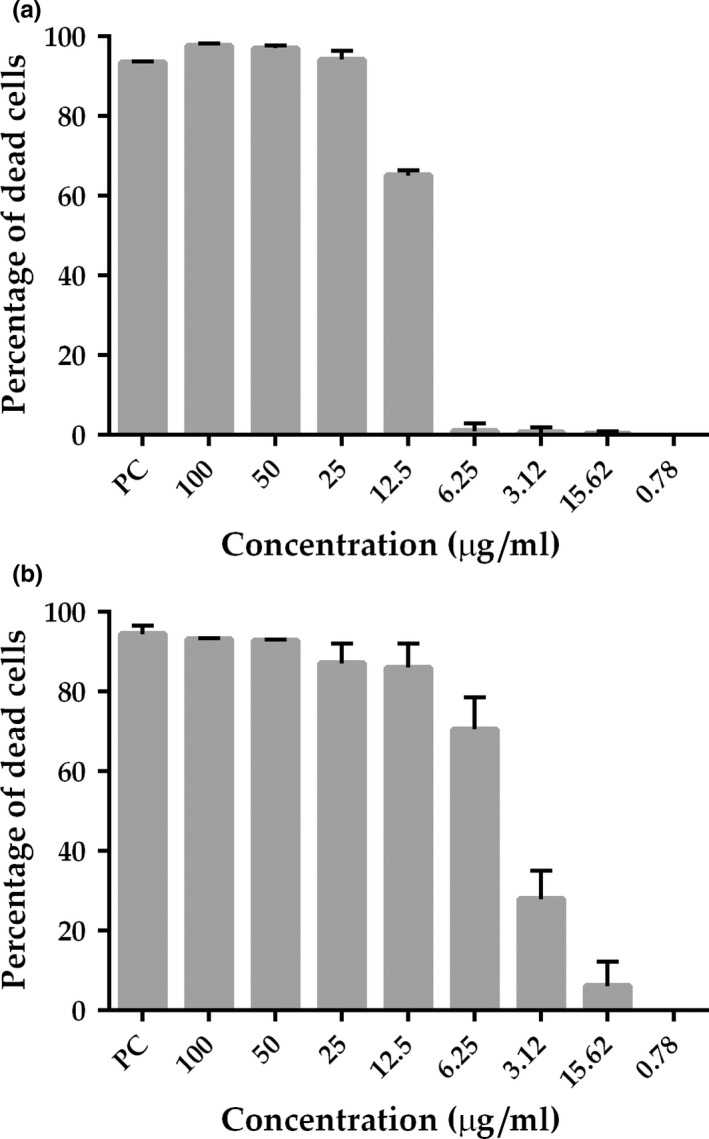
Growth inhibition profile of B. subtilis treated with compounds 1 and 2. Bacterium was exposed to different concentrations of the compounds 1 (a) and 2 (b) (x‐axis) and the percentages of growth inhibition were plotted as vertical bars (y‐axis). Bars represent the average value derived from three independent experiments; standard deviation is depicted above each bar. PC, positive control kanamycin at 20 μg/mL

In order to determine the minimal inhibitory concentration (MIC) of 2 against *B. subtilis*, we exposed this bacterium to various concentrations of the compound and measured the cell respiratory activity using REMA. The concentration window analyzed varied from 100 μg/mL to approximately 0.8 μg/mL. We detected clear concentration–response patterns for both compounds (1 and 2), and the decays of activity started approximately at the concentrations of 12.5 and 6.25 μg/mL, respectively (Figure 3a,b). It was observed that, at least for compound 2 (3B), the following concentrations (~3*–*0.8 μg/mL) induced less inhibition with a soft decline. Subsequently, polynomial regression was used to perform fit to the data, which enabled us to determine the MIC90 of 12.7 μg/mL and MIC50 of 5.4 μg/mL for 2, and the MIC90 of 24.8 μg/mL and MIC50 of 11.5 μg/mL for 1. Therefore, compound 2 displayed a MIC90 that was nearly two times more potent than curcumin, from which it derives. To evaluate if 1 and 2 had bacteriostatic or bactericidal action, aliquots of cell suspensions were collected during REMA and plated in LB medium to check for cell viability. The samples were collected after the exposure to the compounds and before resazurin was added. As a result, colony development was observed for all of the concentrations tested, including those where REMA indicated nearly 100% of cell growth inhibition (above 50 μg/mL). Therefore, both compounds, 1 and 2, have bacteriostatic action against *B. subtilis* at the concentration range analyzed.

### 2.4 Disruption of the divisional septum of *B. subtilis*

**Figure 4:**
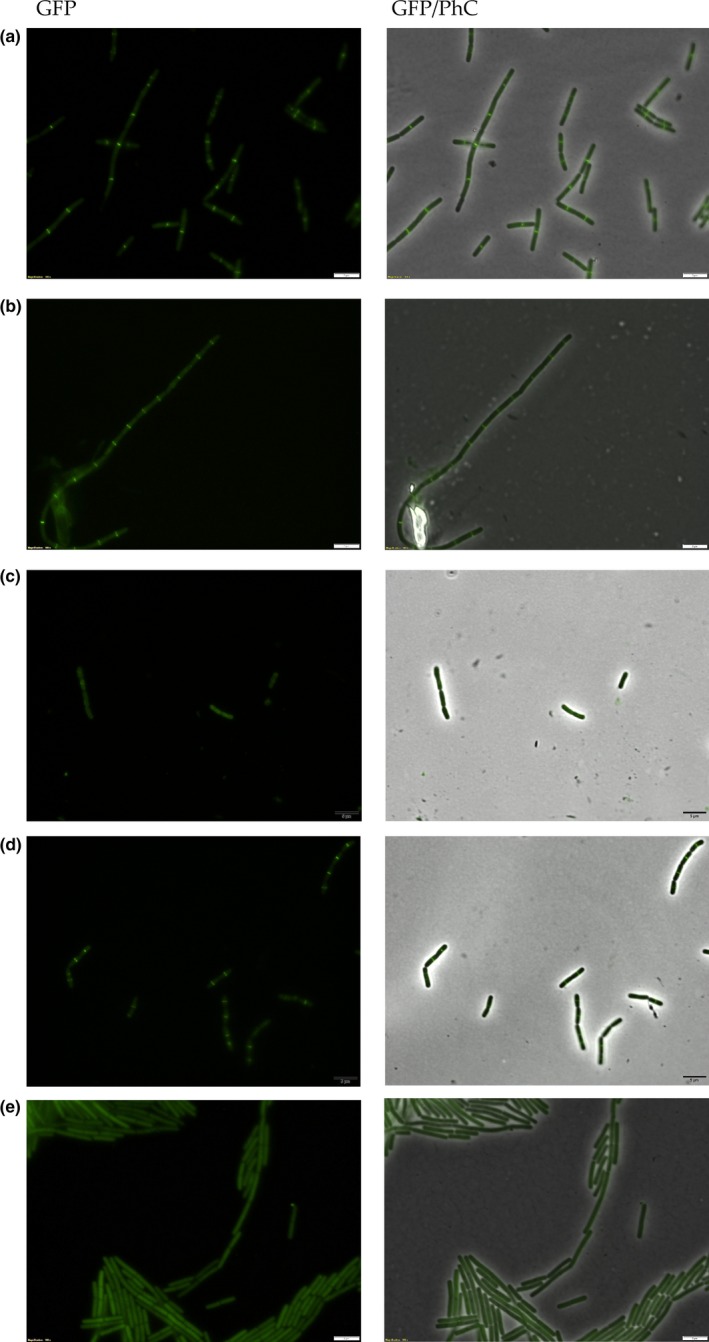
Septum disruption in *B. subtilis*. *B. subtilis* expressing FtsZ‐GFP was cultivated in LB until the OD600 nm of ~0.4; cultures were diluted to 10^5^ cells mL^−1^, and exposed to the test compounds at MIC90. (a) Cells cultivated in LB; (b) LB + 1% DMSO; (c) cells after 15 min of exposure to 1; (d) 30 min of exposure to 2, and (e) 90 min of exposure to 2. GFP/PhC, phase contrast images superimposed on GFP fluorescence images. Magnification 100×; bar, 5 μm

Curcumin (1) has been implicated in the inhibition of the FtsZ‐ring formation in *B. subtilis* (Rai et al., 2008). As 2 is an analogue of 1, we wondered if this compound could perturb the divisional septum of *B. subtilis* as well. To evaluate this, a *B. subtilis* strain expressing FtsZ‐GFP (labeled for the divisional septum) was cultivated in LB, and then diluted in fresh medium so to give a final concentration of 10^5^ cells mL^−1^, which is the same concentration used in REMA. Cells were then exposed to 1 and 2 for up to 30 min, which corresponds to nearly a doubling time of *B. subtilis*, followed by microscopic observations at every 5 min (Figure 4). In panel A (GFP and GFP/PhC merged), we observed the standard pattern of septation (untreated cells), here represented by a bright bar, perpendicular to the long axis of the rods, which is formed by FtsZ‐GFP. The septa here occupy the central portion of the cells under division. For the culture treated with 1% Dimethyl sulfoxide (DMSO), the vehicle in which 1 and 2 were dissolved, the same pattern was documented without any disruption of the divisome (Figure 4b). In agreement with data from Rai et al. (2008), exposure of *B. subtilis* to 1 at MIC90 led to a complete dissolution of the septa after 15 min, with the concomitant accumulation of FtsZ‐GFP fluorescence in the cytoplasm of the cells (Figure 4c). Noteworthy, compound 2 lost the ability to target the divisional septum, even after a longer incubation period of 30 min (Figure 4d). Here the septa are practically identical to the untreated control (compare Figure 4a and d). However, extending the incubation time with 2 for 90 min led to a complete disruption of the septal structures, which was comparable to the treatment with 1 (compare Figure 4c with e). Although 90 min of exposure to the compound may be considered too long, one could argue that 2 may have residual action on septum, specially considering the fact that the cells in panel 4E have no clear sign of morphological damage and/or stress.

### 2.5 Assaying the interference of the simplified curcumin on the FtsZ polymerization dynamics

**Figure 5:**
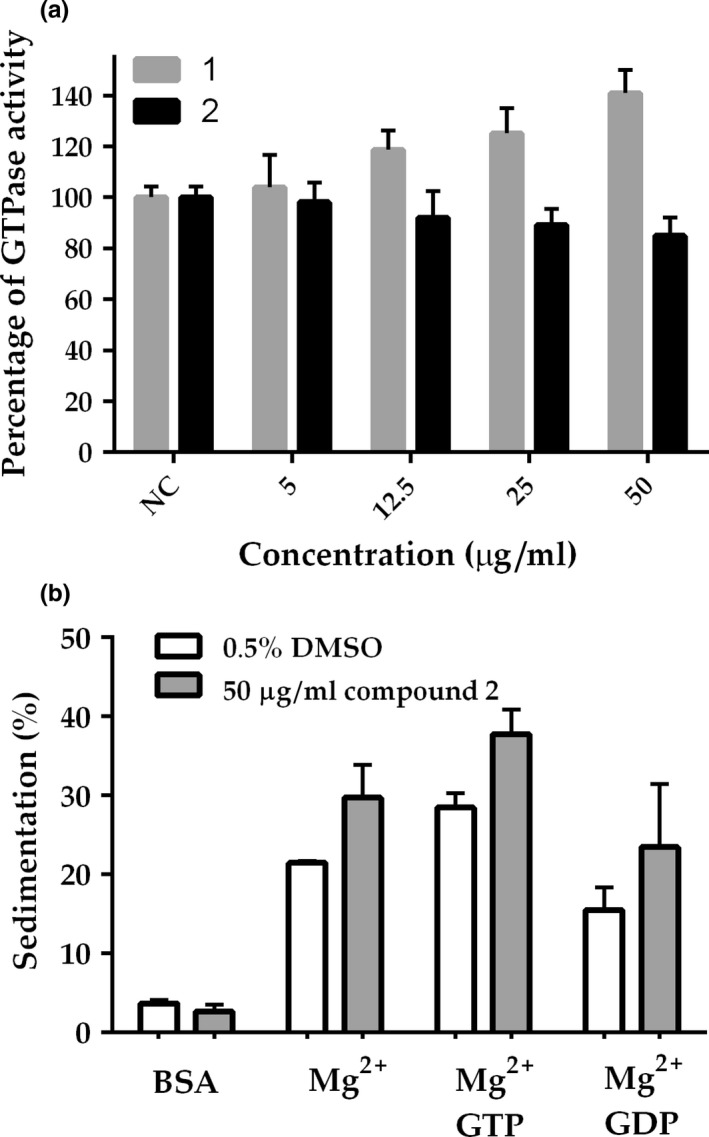
FtsZ GTPase activity and sedimentation dynamics. (a) The GTPase activity of *B. subtilis* FtsZ was evaluated using different concentrations of 1 and 2 (5–50 μg/mL), or the vehicle DMSO at 0.5% (NC). Bars represent average values derived from three independent experiments; standard deviation values are depicted as vertical lines above the bars. (b) Sedimentation of FtsZ: *B. subtilis* FtsZ was incubated with 2 at 50 μg/mL prior to polymerization at 30*°*C, which started by adding nucleotides. Bars represent average values of FtsZ sedimentation (calculated from the pellet fraction in SDS‐PAGE) derived from two independent experiments; standard deviation is depicted above the bars

Disruption of the divisional septum in *B. subtilis* could be a consequence of direct action of 2 on the bacterial tubulin protein FtsZ and/or other proteins or cellular structures that stabilize and modulate the assembly of the septum. FtsZ is a GTPase that polymerizes into protofilaments in the presence of magnesium and both nucleotide cofactors GTP or GDP (Erickson, Anderson, & Osawa, 2010), and is the main scaffold protein of the bacterial septum. The possible interference of 2 was investigated using *B. subtilis* FtsZ and a polymerization/associated GTP hydrolysis assay (Król et al., 2015). Incubation of FtsZ with a concentration of 2 equivalent to the MIC90 (12.7 μg/mL) led to 16% inhibition of the GTPase activity (Figure 5a; compare the black bars at NC and 12.5 μg/mL). When the concentration of 2 was increased fourfold (50 μg/mL), the inhibition doubled and reached 35%, which does not appear to be consistent with a specific action of 2 on the GTPase activity of FtsZ. The same set of experiments was conducted using compound 1, and, in accordance with data from Rai et al. (2008), we documented an increase in the GTPase activity of FtsZ as the concentration of 1 was raised (Figure 5a, gray bars).

Even though 2 does not seem to interfere significantly with the GTPase activity of FtsZ, this compound could somehow preclude FtsZ dynamics by interfering with polymerization and/or bundling of the FtsZ protofilaments. These possibilities were investigated through sedimentation analyses, where FtsZ was exposed to 2 at 50 μg/mL before GTP and GDP were added. Polymerization was initiated by the nucleotide incorporation into the reaction and measured by the determination of the amount of FtsZ recovered in the pellet after centrifugation (Figure 5b). Bovine serum albumin (BSA) was included as a control for aspecific protein aggregation, which would also result in sedimentation. Treatment with 2 and GTP led to a nearly 40% of FtsZ recovered in the pellet. The sedimentation observed with 2 was not significantly different from the sedimentation documented in the presence of DMSO, although 2 did seem to slightly increase the amount of FtsZ recovered. This could be indicative of an increased bundling, that in turn may influence the GTPase activity. BSA did not sediment, indicating that 2 did not cause aspecific protein aggregation.

### 2.6 Simplified curcumin acts on the membrane of *B. subtilis*

**Figure 6:**
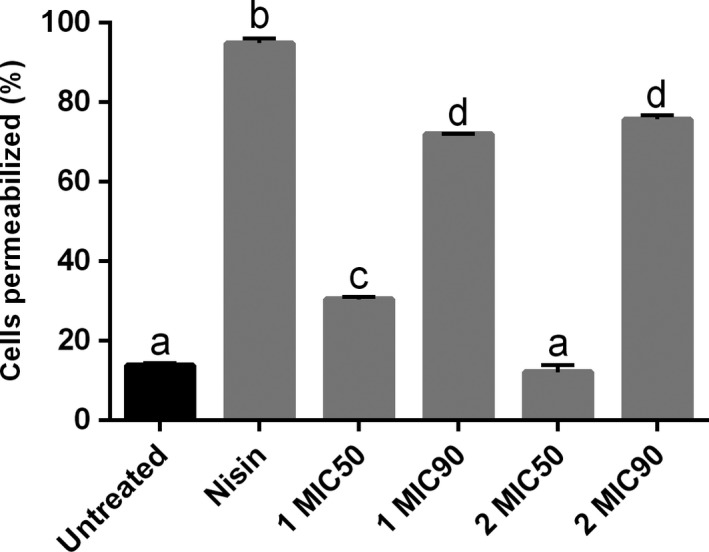
Membrane permeabilization in B. subtilis. Bacterium was exposed to 1 and 2 at MIC50 and MIC90 for 30 min. The percentage of cells with membrane permeabilization is plotted as vertical bars (y‐axis), which represent the average values derived from three independent experiments (in each experiment the number of cells counted was of ~150; n = 450); standard deviation is depicted above each bar. The letters above the bars indicate significant difference among treatments

Tyagi et al. (2015) recently reported that curcumin I, the most active constituent of turmeric, has biological activity against *E. coli*,* E. faecalis*,* S. aureus*, and *P. aeruginosa*, and they also showed that, at least regarding *E. coli* and *S. aureus*, curcumin I exerts its action by disrupting the membrane potential. As membrane potential seems to be necessary for the localization of factors such as FtsZ (Strahl & Hamoen, 2010), compound 2 could be disrupting the *B. subtilis* divisional septum by perturbing its membrane integrity. To collect supportive evidence for this, *B. subtilis* strain 168 cells (10^5^ cells mL^−1^) were exposed to 1 and 2 at MIC50 and MIC90 for 30 min, and stained afterward with the fluorescent dyes SYTO 9 and propidium iodide (PI). These dyes allow the identification of cells with intact and compromised membranes, respectively (Figure 6). In our experiments, untreated cultures normally exhibit ~15% of the cells permeable to PI (untreated). Treatment with 1% DMSO (not shown) essentially resulted in the same pattern observed for the untreated culture. This contrasted sharply with the results of exposure of *B. subtilis* to Nisin (our positive control), a lantibiotic able to form transmembrane pores and to induce high PI intake, affecting almost 100% of the cells (Figure 6). Treatment of the cells with 1 at MIC50 practically doubled the amount of cells compromised when compared with the untreated culture. By increasing the concentration of 1 to MIC90, the extent of cells affected became even more pronounced, reaching ~70% of the cells. Compound 2, on the other hand, had no effect at MIC50, in which the percentage of cells with compromised membranes was practically the same as the untreated. However, by increasing the concentration of 2 to the MIC90, there was a significant increase in PI intake, with the compound affecting ~70% of the cells. Notably, compound 2, at MIC90, was as effective as 1 to induce PI intake in the time‐frame evaluated. In summary, both compounds, 1 and 2, had the ability to target the bacterial membrane, and, judging by the fact that 2 had little influence on the GTPase activity and sedimentation dynamics of purified FtsZ, our data suggest that the primary target of compound 2 is the bacterial membrane.

### 2.7 FT‐IR spectrophotometry

**Figure 7:**
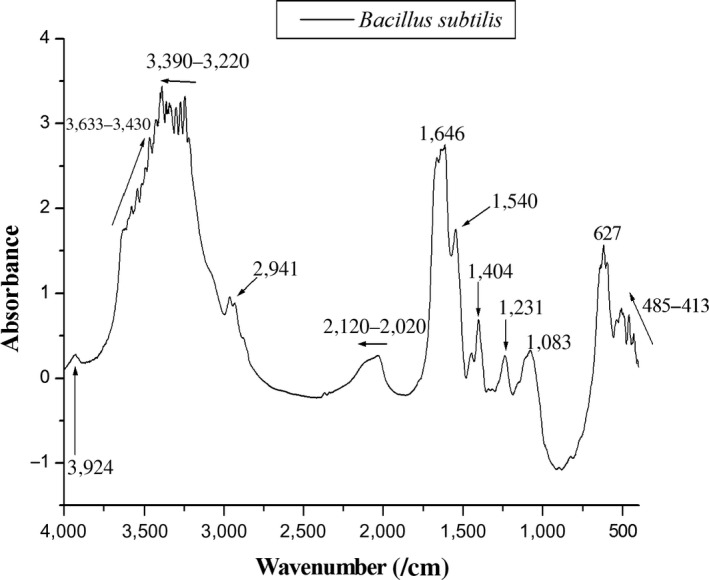
FT‐IR spectrum of untreated cells of *B*. *subtilis*

To investigate the extent of interference 2 might have on the surface of the cells, we employed FT‐IR, a technique potentially able to show perturbations of diverse chemical structures. First, we analyzed the FT‐IR spectrum of untreated *B. subtilis* (Figure 7). According to the literature (Garip, Gozen, & Severcan, 2009), typical bands of the *Bacillus* spectrum could be detected in the regions 1083, 1231, 1540, and 1646 cm^−1^. These peaks represent stretching of C–O bonds found in glycogen (1083 cm^−1^), and –PO^2−^ groups belonging to the structural phospholipids of the cytoplasmic membrane (1231 cm^−1^). The vibrations of N–H bonds and stretching of the C–N bond belonging to the amide II found in alpha helixes of proteins are seen in the 1540 cm^−1^ region, and the stretching of the C=O bond of amide I, also belonging to alpha helixes, is identified at peak 1646 cm^−1^. The peak 1404 cm^−1^ corresponds to vibrations of sulfonic groups present in various structures of the bacterial cell wall. Vibrations in the regions 3390*–*3220 and 3633*–*3430 cm^−1^ are presumed to be residual groups of hydroxyl and amine on the surface of the bacterial biomass. Peaks 2941, 3924, 627, and 485*–*413 cm^−1^ are residues of –CH_3_, O–H, and free amine groups. Finally, the band 2120*–*2020 cm^−1^ corresponds to the CO_2_ from the air. This technique is useful to perform a general reading of the cells, allowing us to mark regions and peaks representing some particular structures. Therefore, any change in a particular peak may be an indication of modification of the original bacterial structure related to that peak (Vishu Kumar, Varadaraj, Gowda, & Tharanathan, 2005).

**Figure 8:**
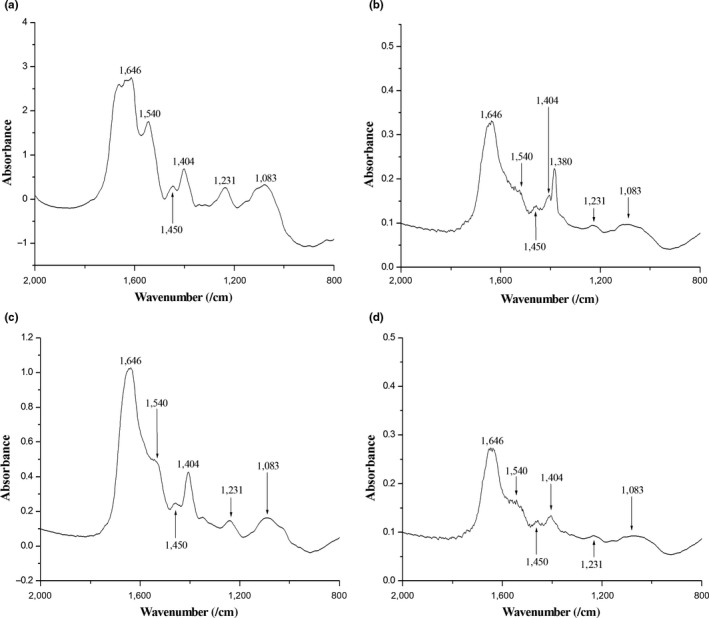
FT‐IR spectra of *B*. *subtilis* exposed to 1 and 2. (a) untreated cells of *B*. *subtilis*; (b) *B*. *subtilis* after contact with 2 at MIC90; (c) 1 at MIC90 and (d) nisin at 5 μg/mL

The region comprised between 4000 and 2000 cm^−1^ represents free amine groups, carbon dioxide from the air, and hydroxyl groups from the bacterial surface. The same is true for the region 800–400 cm^−1^, being also associated with free amine residues and carbon groups from the bacterial surface. For this reason, we concentrated our observations in the spectra ranging from 2000 until 800 cm^−1^ (Figure 8a–d), considered the fingerprint region (Jebsen et al., 2012). When we compared the spectra of *B*. *subtilis* before and after contact with 2 (compare a and b), we identified a decrease in intensity of peaks 1646 and 1540 cm^−1^, which are associated with alpha helix and amide II of proteins. Other peaks that almost disappeared from the spectrum were 1450, 1404, 1231, and 1083 cm^−1^, associated with lipids from the cytoplasmic membrane (1450 cm^−1^), sulfonic groups from the bacterial cell wall (1404 cm^−1^), phospholipids from the cytoplasmatic membrane (1231 cm^−1^), and glycogen (1083 cm^−1^). Exposure to 2 led to the detection of a new peak (1380 cm^−1^) belonging to the vibrations of COO^−^ groups related to fatty acids (Figure 8b). This new finding may be associated to the fragmentation of lipids from the bacterial cell as a consequence of exposure to the compound. The FT‐IR spectrum of *B*. *subtilis* exposed to 1 was very similar in shape to the spectrum of untreated cells (compare a and c), although with an overall lower intensity. This suggests that 1 is not affecting or modifying structures of the bacterial cell that could be captured by this technique, which consequently reinforces the view that the mechanism(s) of action of 1 differ(s) from 2. Noteworthy, the FT‐IR spectrum of *B*. *subtilis* exposed to nisin, known to form pores on the bacterial membrane (Wiedemann, Benz, & Sahl, 2004), displayed changes resembling those induced by 2 (compare a with b and d). The peaks 1646 and 1540 cm^−1^ showed lower intensity as well as noise along the band; the same pattern was seen for peaks 1450 and 1404 cm^−1^. Finally, peaks 1231 and 1083 cm^−1^ virtually disappeared from the spectrum of nisin, a result also observed after exposure to 2 (compare b and d). As the above‐mentioned regions (1646 and 1540, 1646 and 1540, and 1231 and 1083 cm^−1^) are associated with structures mainly found in the cytoplasmic membrane, the FT‐IR data reinforced our suggestion that 2 is targeting the bacterial membrane.

## 3 DISCUSSION

Curcumin (1) is the active constituent of turmeric, a seasoning with a long history of use in popular medicine (Goel, Kunnumakkara, & Aggarwal, 2008). Among the benefits that have been attributed to 1, the antibacterial action was the focus of our studies. Besides, we wanted to investigate the functional group(s) of 1 that are involved with the action it exerts on the bacterial division protein FtsZ (Rai et al., 2008). FtsZ is an ancient tubulin widespread in *Bacteria*. Subunits of FtsZ associate in a GTP‐dependent manner to form polymers, which subsequently bundle to form the Z‐ring, a ring‐like structure that coordinates the bacterial cell division (Hurley et al., 2016; Lutkenhaus, 2007; Xiao & Goley, 2016). In the recent years, FtsZ has become a target for the development of antibacterial compounds. Among the reasons for that we cite its low homology in primary sequence to tubulins of derived eukaryotes, allied to the fact that FtsZ is well conserved throughout *Bacteria* (Hurley et al., 2016; Lock & Harry, 2008; Lowe & Amos, 1998). Such characteristics may represent low(er) toxicity for eukaryotes and larger spectra of action of the FtsZ inhibitors. Rai et al. (2008) reported that 1 inhibits the assembly of FtsZ protofilaments, and stimulates the GTPase activity of the protein. Authors also determined the dissociation constant of 1 to FtsZ (~7 μM), which was considerably low denoting specific binding. In our microscopic analyses, compound 2, a derivative of 1, had only a minor effect on the divisome of *B. subtilis*. In addition, 2 was able to permeabilize the bacterial membrane, an activity also documented for 1 (Figure 6). However, subsequent biochemical characterizations did not support an specific action of 2 on the cell division protein FtsZ. Compound 2 showed only a slightly inhibition of the GTPase activity of *B. subtilis* FtsZ, with a slight interference in FtsZ sedimentation. Our results suggest that disruption of cell division by 2 was probably an indirect consequence of perturbations of the bacterial membrane potential, which is known to interfere with the localization of FtsZ (Strahl & Hamoen, 2010).

To further explore the possible sites of action of 2, *B. subtilis* was exposed to 1 and 2, as well as to nisin, and after that analyzed using FT‐IR. The reasoning here was that FT‐IR, a technique that can potentially show perturbations of several chemical structures, could document alterations induced by the compounds at the surface of the cells. Overall, FT‐IR spectra of the *B. subtilis* exposed to 2 showed reduction in intact cells in the samples. In addition, our results suggest that the perturbation caused by 2 seems to take place mainly at the component structures of the cytoplasmatic membrane. Compound 1 did not induce detectable alterations on the FT‐IR spectrum of *B. subtilis,* which is in agreement with its suggested mode of action: perturbation of the bacterial cell division (Rai et al., 2008). Notably, nisin, a lantibiotic the targets the bacterial membrane producing pores (Wiedemann et al., 2004), induced similar FT‐IR spectral alterations as 2 (Figure 8b,d). Altogether, the FT‐IR spectra were capable of pinpointing chemical structures perturbed in the cells upon exposure to 2. Moreover, the altered regions correspond to chemical structures or cellular components that are part of the cytoplasmatic membrane, seemingly the primary target of 2.

Molecular simplification is a powerful tool that enables the identification of functionality groups within bioactive compounds. This technique applied to 1 seems to have allowed us to discriminate between two of its functionalities: while 1 targets FtsZ and the membrane (Rai et al., 2008; Tyagi et al., 2015), compound 2 targets the membrane more prominently (Figures 5 and 6). In the present work, a set of seven compounds were generated in which the *β*‐diketone of 1 was replaced by a monocarbonyl group (Figure 1), whereas their phenyl rings carry symmetric halogens (–F and –Br) or electron‐donating substituents [–CH_3_, –N(CH_3_)_2_ and –OCH_3_] (Figure 2). Kaur et al. (2010) used *in silico* molecular docking to tentatively identify the interaction between 1 and FtsZ from *E. coli* and *B. subtilis*. Based on their model, the *β*‐diketone portion of 1 interacts with the Glycine residues 21 and 22 of *B. subtilis*‐FtsZ, and with the Glycine residues 20, 21, and 109 of *E. coli*‐FtsZ. Therefore, our results give experimental support for their model in which the presence of the monocarbonyl group replacing the *β*‐diketone may have changed the FtsZ binding properties of 2. In addition, our data shows that the *β*‐diketone group is not required for membrane targeting.

The derived form 2 was further modified into compounds 3*–*8 and their antibacterial properties were assessed using a panel of bacterial isolates of clinical interest, as well as a plant pathogen. The series 2*–*8 was noticeably effective against *S. enterica* sorovar Typhimurium, a model enteropathogenic bacterium that is responsible for acute forms of food‐borne diarrhea. Wotzka, Nguyen, and Hardt (2017) mentioned in their review the need for more efficient therapies against enteropathogens, which include *S. enterica* Typhimurium. In addition to this, acquired antibiotic resistance in *Salmonella* is a matter of concern (Michael & Schwarz, 2016). Here, we described seven compounds (2*–*8) with the ability to inhibit growth of *S. enterica* Typhimurium. Although the maximum inhibition level observed was 75% (7), the anti‐*Salmonella* activity of the series as a whole pointed to the effectiveness of the basic structure of 2 against this bacterium, irrespective of the substituent atom/group used.

Among the compounds, 2 was active against five of seven bacterial isolates. Additionally, 2 showed a nearly 60% increase in potency against *E. faecalis* when compared to 1, while practically keeping the antibacterial property of 1 against *S. enterica* Typhimurium and *B. subtilis*. *E. faecalis* is a gram‐positive bacterium widespread in nature, and also found as a commensal of the gastrointestinal tract of animals. Furthermore, *E. faecalis* has been associated with nosocomial infections and thus poses a considerable threat to human health worldwide. The ability of compound 2 to kill *E. faecalis* emerges as a new therapeutic possibility against an opportunistic pathogen, known to be resilient to several antibiotic treatments (acquired and intrinsic resistance of *E. faecalis* has been nicely reviewed by Guzman Prieto et al., 2016; van Harten, Willems, Martin, & Hendrickx, 2017).

Compound 2 was the only derivative able to kill the plant pathogen *X. citri,* which is the etiological agent of citrus canker, a disease that affects all the commercially important varieties of citrus plants and responsible for significant economic losses to the orange juice industry (Gottwald, Graham, & Schubert, 2002). Citrus canker cannot be treated, however, farmers can manage the problem and control the spread of *X. citri* within the orchards. The current strategy used in Brazil, the major producer of sweet oranges in the world, is the plantation of orange trees that are less susceptible to the pathogen (complete resistance to *X. citri* has not yet been found), the use of wind‐shields (green barriers) to avoid lateral spreading of the bacterium due to the combined action of wind and rains, and the massive use of cupric formulations as a superficial bactericide to protect the aerial parts of the trees (Fundecitrus, Brazil). Copper is toxic to the environment, may be bio/accumulative, and there are reports of acquired resistance of *X. citri* to this metal (Canteros, 1999; Carpene, Andreani, & Isani, 2017). In this sense, the use of agricultural defensives based on less toxic compounds is a desirable alternative that deserves further investment.

## 4 MATERIALS AND METHODS

### 4.1 Synthesis and ^1^H NMR spectra of compounds 2–8

The general procedure for the synthesis of compounds 2–8 was the same as reported by Yadav et al. (2010). Briefly, an amount of 7 mmol acetone was added to a solution of 14 mmol of the respective benzaldehyde in ethanol (10 mL). The solution was stirred at room temperature for 20 min, followed by dropwise addition of ethanolic solution of NaOH (1.5 mL, 1.0 mol/L). The mixture was stirred at room temperature and monitored by successive TLC analyses. When the reaction was finished, the residue was poured into crushed ice. The resulting precipitate of 2 was removed by filtration, washed with cold water, and purified by chromatography over silica gel using mixtures of hexane and ethyl acetate as eluents. Precipitates of 3–8 were removed by filtration, washed with cold water and purified by recrystallization from ethanol. NMR spectra at 25°C were recorded at 600 MHz for ^1^H nucleus on Bruker Avance III spectrometer. Chemical shifts are given in ppm on the δ scale referenced to residual CHCl_3_ signal at 7.28, and coupling constants (*J*) are calculated in hertz (Hz).

### 4.2 Strains and growth conditions

The strains *Enterococcus faecalis* ATCC29212 (*E. faecalis*), *Staphylococcus aureus* ATCC25923 (*S. aureus*), *Escherichia coli* ATCC25922 (*E. coli*), *Salmonella enterica* sorovar Typhimurium ATCC13311 (*S. enterica*), and *Pseudomonas aeruginosa* ATCC27853 (*P. aeruginosa*) were obtained from Fiocruz (Fundação Oswaldo Cruz, Rio de Janeiro, Brazil). *Bacillus subtilis* 168 (*B. subtilis*), and *B. subtilis* expressing FtsZ‐GFP (*amy*::*pspac‐ftsZ‐gfpmut1*) were gifts from Frederico Gueiros‐Filho (Dept. of Biochemistry, IQ, Universidade de São Paulo, Brazil). *Xanthomonas citri* subsp. *citri* 306 (*X. citri)* was obtained from the Instituto Biológico, Seção de Bactérias Fitopatológicas (IBSBF‐1594; Schaad et al., 2006). *B. subtilis* was cultivated in LB/LB‐agar (Sambrook, Fritsch, & Maniatis, 1989) at 30°C; *E. coli* in Tryptic Soy Broth (TSB) or TSB‐agar (Difco‐211225) at 37°C; *X. citri* was cultivated in NYG/NYG‐agar (Peptone 5 g, yeast extract 3 g and glycerol 20 g in 1.0 liter of distilled water) at 30°C; *E. faecalis* in Brain Heart Infusion (BHI) or BHI‐agar (Difco‐237500) at 37°C; *P. aeruginosa* and *S. enterica* were cultivated in Nutrient Broth (NB) or NB‐agar (Difco‐234000) at 37°C.

### 4.3 Cell viability tests

The antibacterial activity of the compounds was evaluated using the Resazurin Microtiter Assay Plate (REMA) adapted to *X. citri* (Silva et al., 2013). Compounds, as dried powders, were diluted in 100% DMSO to a starting concentration of 10 mg/mL. Following, compounds were diluted in a twofold scheme using growth medium (according to the medium recommended for each strain), and aliquots were deposited directly into the wells of a 96‐microtiter plate so to give the necessary concentration to be tested in a final volume of 100 μL. Cells were standardized to 10^5^ CFU/well. The negative control was media; the vehicle control was 1% DMSO, and as positive controls we used 20 μg/mL of kanamycin for *X. citri* and *B. subtilis,* 8 μg/mL of ampicillin for *E. coli* and *S. typhimurium*, 4 μg/mL of gentamicin for *P. aeruginosa*, and 2 μg/mL and 4 μg/mL of vancomycin for *S. aureus* and *E. faecalis*, respectively (EUCAST/BrCAST, 2017). Bacteria were exposed to the compounds for a period of 12 hr. After the incubation time with the compounds, the metabolic activity of the cells was assessed by adding resazurin (0.1 mg/mL) to the samples to the final concentration of 1.5 μg/mL, followed by a further incubation of ~2 hr. Resazurin is a nonfluorescent blue dye that can be reduced in the presence of NADH to a reddish fluorescent compound known as resorufin (an indication of cell metabolism). The amount of resazurin reduced by NADH to its fluorescent form is quantifiable in REMA, and it is directly proportional to the amount of cells with active metabolism (respiring); therefore, we can measure the proportion of the cells in a culture that are affected by a specific compound or treatment. Fluorescence scanning was conducted using a microplate reader Synergy H1 (Biotek). Three independent experiments were used to estimate the minimal inhibitory concentration (MIC) data. Statistics were carried out using Graphpad Prism version 6. To evaluate if compounds had bacteriostatic or bactericidal action, samples from REMA were plated on agar‐media and incubated for 24 hr (48 hr for *X. citri*) in order to check for the ability of the cells to grow.

### 4.4 Microscopy

Cells to be evaluated under the microscope were collected from REMA plates after the exposure to the compounds and immobilized in agarose‐covered slides essentially as described by Martins et al. (2010). *B. subtilis* labeled for the septum was cultivated in the presence of 0.02 mM Isopropyl β‐D‐thiogalactopyranoside (IPTG) to induce the expression of FtsZ‐GFP from the *pspac* promoter. Membrane integrity was assessed using the Live/Dead Baclight kit (Thermo‐Scientific L7012) following the exposure to the compounds in REMA. All the visualizations were done using an Olympus BX‐61 microscope, equipped with a monochromatic OrcaFlash‐2.8 camera, and guided by the software CellSens version 11.

### 4.5 FtsZ assays

*B. subtilis* FtsZ was expressed and purified using the ammonium sulfate precipitation method as described before (Krol & Scheffers, 2013; Mukherjee & Lutkenhaus, 1998). The FtsZ GTP hydrolysis rate was determined using the malachite green phosphate assay as described in (Król et al., 2015). Twelve μM FtsZ was incubated with 10 mM MgCl_2_ in the presence of various concentrations of 2 in 50 mM HEPES pH 7.5, 300 mM KCl at 30°C and the reaction was started with 1 mM GTP. As a control, 1% DMSO was used. FtsZ sedimentation assays were performed as described in (Król et al., 2015). Ten μM FtsZ and BSA (control) were incubated with 10 mM MgCl_2_ in the presence of 50 μg/mL of 2 0.5% DMSO (control) in 50 mM HEPES pH 7.5, 50 mM KCl at 30°C and the reaction was started with 2 mM GTP or GDP (or a corresponding volume of buffer added where no nucleotide was present).

### 4.6 FT‐IR spectrophotometry

FT‐IR spectroscopy was performed according Zeroual, Manfait, and Choisy (1995) with some modifications. Cells were analyzed before and after treatment with the compounds using the FT‐IR spectrophotometer Shimadzu, Model 8300. Briefly, bacteria were cultivated in liquid medium up to the concentration of 10^5^ cells/mL; after growth, cells were centrifuged at 10,000 *g* for 2 min in order to form pellets at the bottom of 1.5 mL microcentrifuge tubes. Cells were dissolved in deionized water, centrifuged and then washed two more times in water to remove traces of medium. After that, cell mass was mixed together with 149 mg of KBr. To obtain the FT‐IR spectra of treated cells, compounds were added to the cultures at MIC90 and allowed to react for 30 min before the centrifugation steps. Nisin was added as a control for membrane pore formation at 5 μg/mL. The dry samples with KBr were homogenized and compressed at 40 kN for 5 min in preparation for the FT‐IR readings. Absorbance was analyzed over a range of 400 to 4000 cm^*−*1^, with 32 scans at a resolution of 4 cm^*−*1^. Data treatment and analyses were performed using the software Origin 8.00.

## CONFLICT OF INTEREST

The authors declare no conflict of interest.

## References

[mbo3683-bib-0001] Canteros, B. I. (1999). Copper resistance in xanthomonas campestris pv. Citri In Mahadevan A. (Ed.), Plant pathogenic bacteria. Proceedings of the international society of bacteriology, centre for advanced study in botany (pp. 455–459). Chennai, India: University of Madras.

[mbo3683-bib-0002] Carpene, E. , Andreani, G. , & Isani, G. (2017). Trace elements in unconventional animals: A 40‐year experience. Journal of Trace Elements in Medicine and Biology: Organ of the Society for Minerals and Trace Elements, 43, 169–179. 10.1016/j.jtemb.2017.02.003 28215718

[mbo3683-bib-0003] Chen, J. , He, Z. M. , Wang, F. L. , Zhang, Z. S. , Liu, X. Z. , Zhai, D. D. , & Chen, W. D. (2016). Curcumin and its promise as an anticancer drug: An analysis of its anticancer and antifungal effects in cancer and associated complications from invasive fungal infections. European Journal of Pharmacology, 772, 33–42. 26723514 10.1016/j.ejphar.2015.12.038

[mbo3683-bib-0004] Di Martino, R. M. , Luppi, B. , Bisi, A. , Gobbi, S. , Rampa, A. , Abruzzo, A. , & Belluti, F. (2017). Recent progress on curcumin‐based therapeutics: A patent review (2012–2016). Part i: Curcumin. Expert Opinion on Therapeutic Patents, 27, 579–590. 10.1080/13543776.2017.1276566 28024125

[mbo3683-bib-0005] Erickson, H. P. , Anderson, D. E. , & Osawa, M. (2010). FtsZ in bacterial cytokinesis: Cytoskeleton and force generator all in one. Microbiology and Molecular Biology Reviews, 74, 504–528. 10.1128/MMBR.00021-10 21119015 PMC3008173

[mbo3683-bib-0006] Garip, S. , Gozen, A. C. , & Severcan, F. (2009). Use of fourier transform infrared spectroscopy for rapid comparative analysis of bacillus and micrococcus isolates. Food Chemistry, 113, 1301–1307. 10.1016/j.foodchem.2008.08.063

[mbo3683-bib-0007] Goel, A. , Kunnumakkara, A. B. , & Aggarwal, B. B. (2008). Curcumin as “curecumin”: From kitchen to clinic. Biochemical Pharmacology, 75, 787–809. 10.1016/j.bcp.2007.08.016 17900536

[mbo3683-bib-0008] Gordh, T. (2010). Lidocaine: The origin of a modern local anesthetic. 1949. Anesthesiology, 113, 1433–1437. 10.1097/ALN.0b013e3181fcef48 21068652

[mbo3683-bib-0009] Gottwald, T. R. , Graham, J. H. , & Schubert, T. S. (2002). Citrus canker: The pathogen and its impact. Plant Health Progress, 10, 32 10.1094/PHP-2002-0812-01-RV

[mbo3683-bib-0010] Grigg, M. J. , William, T. , Menon, J. , Barber, B. E. , Wilkes, C. S. , Rajahram, G. S. , … Anstey, N. M. (2016). Efficacy of artesunate‐mefloquine for chloroquine‐resistant plasmodium vivax malaria in Malaysia: An open‐label, randomized, controlled trial. Clinical Infectious Diseases: An Official Publication of the Infectious Diseases Society of America, 62, 1403–1411. 10.1093/cid/ciw121 27107287 PMC4872287

[mbo3683-bib-0011] Guzman Prieto, A. M. , van Schaik, W. , Rogers, M. R. , Coque, T. M. , Baquero, F. , Corander, J. , & Willems, R. J. (2016). Global emergence and dissemination of enterococci as nosocomial pathogens: Attack of the clones? Frontiers in Microbiology, 7, 788. 27303380 10.3389/fmicb.2016.00788 PMC4880559

[mbo3683-bib-0012] Hampannavar, G. A. , Karpoormath, R. , Palkar, M. B. , & Shaikh, M. S. (2016). An appraisal on recent medicinal perspective of curcumin degradant: Dehydrozingerone (dzg). Bioorganic & Medicinal Chemistry, 24, 501–520. 10.1016/j.bmc.2015.12.049 26796952

[mbo3683-bib-0013] Heger, M. , van Golen, R. F. , Broekgaarden, M. , & Michel, M. C. (2014). The molecular basis for the pharmacokinetics and pharmacodynamics of curcumin and its metabolites in relation to cancer. Pharmacological Reviews, 66, 222–307. 24368738 10.1124/pr.110.004044

[mbo3683-bib-0014] Hung, W. C. , Chen, F. Y. , Lee, C. C. , Sun, Y. , Lee, M. T. , & Huang, H. W. (2008). Membrane‐thinning effect of curcumin. Biophysical Journal, 94, 4331–4338. 10.1529/biophysj.107.126888 18310254 PMC2480666

[mbo3683-bib-0015] Hurley, K. A. , Santos, T. M. , Nepomuceno, G. M. , Huynh, V. , Shaw, J. T. , & Weibel, D. B. (2016). Targeting the bacterial division protein ftsz. Journal of Medicinal Chemistry, 59, 6975–6998. 10.1021/acs.jmedchem.5b01098 26756351 PMC13006086

[mbo3683-bib-0016] Ingolfsson, H. I. , Koeppe, R. E. 2nd , & Andersen, O. S. (2007). Curcumin is a modulator of bilayer material properties. Biochemistry, 46, 10384–10391. 10.1021/bi701013n 17705403

[mbo3683-bib-0017] Janssen, P. A. J. , & van der Eycken, C. A. M. (1968). The chemical anatomy of potent morphine‐like analgesics, in drugs affecting the central nervous system (pp. 25–60). New York: Marcel Dekker.

[mbo3683-bib-0018] Jebsen, C. , Norici, A. , Wagner, H. , Palmucci, M. , Giordano, M. , & Wilhelm, C. (2012). Ftir spectra of algal species can be used as physiological fingerprints to assess their actual growth potential. Physiologia Plantarum, 146, 427–438. 10.1111/j.1399-3054.2012.01636.x 22540209

[mbo3683-bib-0019] Kaur, S. , Modi, N. H. , Panda, D. , & Roy, N. (2010). Probing the binding site of curcumin in escherichia coli and *Bacillus subtilis* FtsZ–a structural insight to unveil antibacterial activity of curcumin. European Journal of Medicinal Chemistry, 45, 4209–4214. 10.1016/j.ejmech.2010.06.015 20615583

[mbo3683-bib-0020] Król, E. , de Sousa Borges, A. , da Silva, I. , Polaquini, C. R. , Regasini, L. O. , Ferreira, H. , & Scheffers, D. J. (2015). Antibacterial activity of alkyl gallates is a combination of direct targeting of FtsZ and permeabilization of bacterial membranes. Frontiers in Microbiology, 6, 1–12. 25972861 10.3389/fmicb.2015.00390 PMC4413848

[mbo3683-bib-0021] Krol, E. , & Scheffers, D. J. (2013). FtsZ polymerization assays: Simple protocols and considerations. Journal of Visualized Experiments, 16, e50844 10.3791/50844 PMC3991336 24300445

[mbo3683-bib-0022] Kumar, A. , Dhamgaye, S. , Maurya, I. K. , Singh, A. , Sharma, M. , & Prasad, R. (2014). Curcumin targets cell wall integrity via calcineurin‐mediated signaling in Candida albicans. Antimicrobial Agents and Chemotherapy, 58, 167–175. 10.1128/AAC.01385-13 24145527 PMC3910804

[mbo3683-bib-0023] Lock, R. L. , & Harry, E. J. (2008). Cell‐division inhibitors: New insights for future antibiotics. Nature Reviews Drug Discovery, 7, 324–338. 10.1038/nrd2510 18323848

[mbo3683-bib-0024] Lowe, J. , & Amos, L. A. (1998). Crystal structure of the bacterial cell‐division protein FtsZ. Nature, 391, 203–206. 10.1038/34472 9428770

[mbo3683-bib-0025] Lutkenhaus, J. (2007). Assembly dynamics of the bacterial mincde system and spatial regulation of the z ring. Annual Review of Biochemistry, 76, 539–562. 10.1146/annurev.biochem.75.103004.142652 17328675

[mbo3683-bib-0026] Martins, P. M. , Lau, I. F. , Bacci, M. , Belasque, J. , do Amaral, A. M. , Taboga, S. R. , & Ferreira, H. (2010). Subcellular localization of proteins labeled with GFP in *Xanthomonas citri* ssp. *citri*: targeting the division septum. FEMS Microbiology Letters, 310, 76–83. 20629754 10.1111/j.1574-6968.2010.02047.x

[mbo3683-bib-0027] Michael, G. B. , & Schwarz, S. (2016). Antimicrobial resistance in zoonotic nontyphoidal salmonella: An alarming trend? Clinical Microbiology and Infection: The Official Publication of the European Society of Clinical Microbiology and Infectious Diseases, 22, 968–974. 10.1016/j.cmi.2016.07.033 27506509

[mbo3683-bib-0028] Moghadamtousi, S. Z. , Kadir, H. A. , Hassandarvish, P. , Tajik, H. , Abubakar, S. , & Zandi, K. (2014). A review on antibacterial, antiviral, and antifungal activity of curcumin. BioMed Research International, 2014, 186864. 24877064 10.1155/2014/186864 PMC4022204

[mbo3683-bib-0029] Mukherjee, A. , & Lutkenhaus, J. (1998). Purification, assembly, and localization of FtsZ. Methods in Enzymology, 298, 296–305. 10.1016/S0076-6879(98)98026-0 9751889

[mbo3683-bib-0030] Murray, M. C. , & Perkins, M. E. (1996). Chemotherapy of malaria. Annual Reports in Medicinal Chemistry, 31, 141–150. 10.1016/S0065-7743(08)60454-6

[mbo3683-bib-0031] Nelson, K. M. , Dahlin, J. L. , Bisson, J. , Graham, J. , Pauli, G. F. , & Walters, M. A. (2017). The essential medicinal chemistry of curcumin. Journal of Medicinal Chemistry, 60, 1620–1637. 10.1021/acs.jmedchem.6b00975 28074653 PMC5346970

[mbo3683-bib-0032] Rai, D. , Singh, J. K. , Roy, N. , & Panda, D. (2008). Curcumin inhibits FtsZ assembly: An attractive mechanism for its antibacterial activity. The Biochemical Journal, 410, 147–155. 10.1042/BJ20070891 17953519

[mbo3683-bib-0033] Sambrook, J. , Fritsch, E. F. , & Maniatis, T. (1989). Molecular cloning: A laboratory manual (pp 626). Cold Spring Harbor, NY: Cold Spring Harbor Laboratory.

[mbo3683-bib-0034] Schaad, N. W. , Postnikova, E. , Lacy, G. , Sechler, A. , Agarkova, I. , Stromberg, P. E. , … Vidaver, A. K. (2006). Emended classification of xanthomonad pathogens on citrus. Systematic and Applied Microbiology, 29, 690–695. 10.1016/j.syapm.2006.08.001 17183629

[mbo3683-bib-0035] Silva, I. C. , Regasini, L. O. , Petronio, M. S. , Silva, D. H. , Bolzani, V. S. , Belasque, J. Jr , … Ferreira, H. (2013). Antibacterial activity of alkyl gallates against *Xanthomonas citri* subsp. *citri* . Journal of Bacteriology, 195, 85–94. 10.1128/JB.01442-12 23104804 PMC3536167

[mbo3683-bib-0036] Strahl, H. , & Hamoen, L. W. (2010). Membrane potential is important for bacterial cell division. Proceedings of the National Academy of Sciences of the United States of America, 107, 12281–12286. 10.1073/pnas.1005485107 20566861 PMC2901462

[mbo3683-bib-0037] Tyagi, P. , Singh, M. , Kumari, H. , Kumari, A. , & Mukhopadhyay, K. (2015). Bactericidal activity of curcumin i is associated with damaging of bacterial membrane. PLoS ONE, 10, 1–15. 10.1371/journal.pone.0121313 PMC4374920 25811596

[mbo3683-bib-0038] Valli, M. , Altei, W. , dos Santos, R. N. , de Lucca, E. C. Jr. , Dessoy, M. A. , Pioli, R. M. , … Dias, L. C. (2017). Synthetic analogue of the natural product piperlongumine as a potent inhibitor of breast cancer cell line migration. Journal of the Brazilian Chemical Society, 28, 475–484. 10.21577/0103-5053.20160303

[mbo3683-bib-0039] van Harten, R. M. , Willems, R. J. , Martin, N. I. , & Hendrickx, A. P. (2017). Multidrug‐resistant enterococcal infections: New compounds, novel antimicrobial therapies? Trends in Microbiology, 25, 467–479. 10.1016/j.tim.2017.01.004 28209400

[mbo3683-bib-0040] Vishu Kumar, A. B. , Varadaraj, M. C. , Gowda, L. R. , & Tharanathan, R. N. (2005). Characterization of chito‐oligosaccharides prepared by chitosanolysis with the aid of papain and pronase, and their bactericidal action against *Bacillus cereus* and *Escherichia coli* . Biochemical Journal, 391, 167–175. 10.1042/BJ20050093 15932346 PMC1276913

[mbo3683-bib-0041] Wiedemann, I. , Benz, R. , & Sahl, H. G. (2004). Lipid II‐mediated pore formation by the peptide antibiotic nisin: A black lipid membrane study. Journal of Bacteriology, 186, 3259–3261. 10.1128/JB.186.10.3259-3261.2004 15126490 PMC400633

[mbo3683-bib-0042] Wotzka, S. Y. , Nguyen, B. D. , & Hardt, W. D. (2017). Salmonella Typhimurium diarrhea reveals basic principles of enteropathogen infection and disease‐promoted DNA exchange. Cell Host & Microbe, 21, 443–454. 10.1016/j.chom.2017.03.009 28407482

[mbo3683-bib-0043] Xiao, J. , & Goley, E. D. (2016). Redefining the roles of the ftsz‐ring in bacterial cytokinesis. Current Opinion in Microbiology, 34, 90–96. 10.1016/j.mib.2016.08.008 27620716 PMC5164845

[mbo3683-bib-0044] Yadav, B. , Taurin, S. , Rosengren, R. J. , Schumacher, M. , Diederich, M. , Somers‐Edgar, T. J. , & Larsen, L. (2010). Synthesis and cytotoxic potential of heterocyclic cyclohexanone analogues of curcumin. Bioorganic & Medicinal Chemistry, 18, 6701–6707. 10.1016/j.bmc.2010.07.063 20728364

[mbo3683-bib-0045] Zeroual, W. , Manfait, M. , & Choisy, C. (1995). FT‐IR spectroscopy study of perturbations induced by antibiotic on bacteria (*Escherichia coli*). Pathologie‐Biologie, 43, 300–305. 7567119

